# In ovo Injection of a Galacto-Oligosaccharide Prebiotic in Broiler Chickens Submitted to Heat-Stress: Impact on Transcriptomic Profile and Plasma Immune Parameters

**DOI:** 10.3390/ani9121067

**Published:** 2019-12-02

**Authors:** Micol Bertocchi, Marco Zampiga, Diana Luise, Marika Vitali, Federico Sirri, Anna Slawinska, Siria Tavaniello, Orazio Palumbo, Ivonne Archetti, Giuseppe Maiorano, Paolo Bosi, Paolo Trevisi

**Affiliations:** 1 Department of Agricultural and Food Sciences (DISTAL), University of Bologna, 40126 Bologna, Italy; micol.bertocchi2@unibo.it (M.B.); marco.zampiga2@unibo.it (M.Z.); diana.luise2@unibo.it (D.L.); marika.vitali4@unibo.it (M.V.); federico.sirri@unibo.it (F.S.); paolo.trevisi@unibo.it (P.T.); 2 Department of Agricultural, Environmental and Food Sciences, University of Molise, 86100 Campobasso CB, Italy; siria.tavaniello@unimol.it (S.T.); maior@unimol.it (G.M.); 3 Department of Animal Biotechnology and Genetics, UTP University of Science and Technology, 85-084 Bydgoszcz, Poland; slawinska@utp.edu.pl; 4 Division of Medical Genetics, Fondazione IRCCS Casa Sollievo della Sofferenza, 71013 San Giovanni Rotondo (FO), Italy; palumboorazio.op@gmail.com; 5 Laboratory of Animal Welfare, Clinical Biochemistry and Veterinary Immunology, Istituto Zooprofilattico Sperimentale della Lombardia e dell’Emilia Romagna Bruno Ubertini, 25124 Brescia, Italy; ivonne.archetti@izsler.it

**Keywords:** galactooligosaccharides, heat stress, *in ovo* injection, intestine, transcriptome

## Abstract

**Simple Summary:**

Galactooligosaccharides (GOS) delivered *in ovo* stimulate development of indigenous microflora in the chicken embryo. Such stimulation may create a eubiotic environment in the guts which positively influences intestinal function and health. This study aimed to evaluate the impact of GOS delivered *in ovo* on the modulation of the transcriptomic responses in the intestinal tissues of broiler chickens challenged (or not challenged) with heat in the last growing phase. GOS stimulated several groups of genes in jejunum and in cecum independently of the heat stress provision. A general favourable effect of GOS was recognized due to the enrichment of energetic metabolism-related gene sets, mainly in jejunum. The enrichment of lipid metabolism-related gene sets in the GOS group might have contributed to gut function and barrier maintenance, which might also be linked to a reduced immune system activation, mainly at cecum level. The heat stress impaired gut functions in terms of energy and immunity, in agreement with previous studies. Under heat stress condition, the *in ovo* injection of GOS did not provide additional benefits to the intestinal transcriptomic response.

**Abstract:**

This study investigated the effects of a galactooligosaccharide (GOS) prebiotic *in ovo* injected on intestinal transcriptome and plasma immune parameters of broiler chickens kept under thermoneutral (TN) or heat stress (HS) conditions. Fertilized Ross 308 eggs were injected *in ovo* with 0.2 mL physiological saline without (control, CON) or with 3.5 mg of GOS (GOS). Three-hundred male chicks/injection treatment (25 birds/pen) were kept in TN or HS (30 °C) conditions during the last growing phase, in a 2 × 2 factorial design. At slaughter, from 20 birds/injection group (half from TN and half from HS), jejunum and cecum were collected for transcriptome analysis, and plasma was collected. No differences in plasma parameters (IgA and IgG, serum amyloid) and no interaction between injection treatment and environment condition were found. GOS-enriched gene sets related to energetic metabolism in jejunum, and to lipid metabolism in cecum, were involved in gut barrier maintenance. A homogeneous reaction to heat stress was determined along the gut, which showed downregulation of the genes related to energy and immunity, irrespective of *in ovo* treatment. GOS efficacy in counteracting heat stress was scarce after ten days of environmental treatment, but the *in ovo* supplementation modulates group of genes in jejunum and cecum of broiler chickens.

## 1. Introduction

Genetic selection for fast-growing and heavy hybrid broilers has halved the duration of rearing [1,2]. At the same time, an increasing incidence of stress-induced myopathies and environmental stress susceptibility have been observed [1,3]. Currently, heat stress represents the main environmental factor negatively affecting poultry production [4]. Fast-growing broilers are highly susceptible to heat stress due to the low thermoregulatory capacity, compared to unimproved fowls [5]. The low heat loss capacity, which is partially influenced by the body size, results in thermoregulatory effort with respiratory breathlessness that requires a high metabolic cost, which in turn increases heat production [6]. Besides the impact on meat quality, heat stress strongly affects animal health by inducing oxidative damage to lipid, protein and DNA, leading to endocrine disorders and negative consequences on immune response and increasing inflammatory cytokines and intestinal dysfunction [7,8]. At the intestinal level, heat stress alters the microbial ecology and gut epithelium with a possible onset of enteric diseases [9,10]. Commensal gut bacteria protect the host from pathogens by stimulating immune responses, competing for epithelial binding sites and producing bacteriocins.

At the intestinal level, supplementations of probiotics in chicken under heat stress conditions have been shown to enhance beneficial gut bacteria and improve intestinal micro-architecture [11,12,13]. Galactooligosaccharides (GOS) are fermentable oligosaccharides not digested by the host that can act as a prebiotic when selectively stimulating growth of beneficial bacteria in the gut [14]. In chicken, GOS dietary supplementation selectively stimulated the gut microbiota with increased faecal total anaerobic bacteria, lactobacilli and bifidobacteria [15], and improved gut barrier and gut-associated immunity through stimulation of cecal tonsil gene expression [16]. Dietary GOS also prevented the heat stress-related changes in jejunum, but not in the ileum of broilers [17].

In order to achieve the desired efficacy of gut maturation, prebiotics must be administered to an animal as early in life as possible. Conventionally, in-feed or in-water supplementation has been used during the first hours/day post-hatching. However, this approach relies on the amount of feed and/or water intake, quality of water (chlorinated) and other experimental factors [18]. As an alternative, an *in ovo* approach for injection of prebiotics directly to the incubating egg has been developed [19]. It allows for a precise delivery of the bioactive substance to all embryos at the early stage of development, which assures proper development of gut microbial population across the flock. *In ovo* injection at the 12^th^ day of embryonic development was demonstrated to stimulate gut co-development, gut-associated lymphoid tissue (GALT) and microbiota, with a beneficial effect on the post-hatching development of chickens [19,20,21,22,23]. Moreover, a single *in ovo* injection allows a long-term regulation of immune-related molecular pathways with the development of adaptive immune response [24], a maintenance of *Bifidobacteria* throughout the growing period, and a stimulation of *Lactobacillus* species and *Bifidobacteria* even at very low doses [19,20,25]. Given these previous results, the *in ovo* technology can be considered the best solution for bioactive compounds delivery, to ensure the protection of gastrointestinal tract [26].

Despite the positive results on heat stress mitigation obtained following *in ovo* injection with GOS [27], the knowledge of the involved molecular and biological processes is scarce. Transcriptomics is, therefore, a valid method to identify biological patterns underlying changes in the intestines of chickens. According to that, a recent study reported that *in ovo* injected GOS prebiotic induced a decrease in splenic RNA expression of antioxidative genes and of a heat-shock protein gene in broiler chickens challenged with short-term heat stress [28].

Considering the hypothesis of a mitigation of heat environmental stress through the *in ovo* injection of GOS prebiotic, the main aim of this study was to investigate the effect of *in ovo* injection of GOS on the intestinal transcriptomic profile of broiler chicken kept under thermoneutral conditions or under chronic heat stress conditions during the last phase of rearing period. Moreover, the study aimed also to investigate the plasma immune parameters of broiler chicken in order to assess, for the first time, any physiological immune imbalance that may occur following GOS *in ovo* injection under normal or heat stress conditions.

## 2. Materials and Methods

### 2.1. Animals Tested, Experimental Groups and Overall Sampling

The experimental design was approved by the Italian Ministry of Health with protocol number 503/2016. The experiment was carried out based on 2 × 2 factorial design with GOS *in ovo* injection and chronic heat stress as factors. Overall, these treatments resulted in a total of four experimental groups: Control group with thermoneutral condition (CON/TN); control group under heat stress in the last 10 days of growing (CON/HS); GOS *in ovo* group with thermoneutral condition (GOS/TN); GOS *in ovo* group under heat stress the last 10 days of rearing (GOS/HS).

Fertilized eggs of broiler chickens (Ross 308, 2000 eggs) were incubated in a commercial hatchery following the standard procedure. On day 12 of egg incubation, a single dose of 3.5 mg GOS dissolved in 0.2 mL of 0.9% physiological saline (0.9% NaCl) (referred to hereafter as GOS) was inoculated into the air cell using a needle syringe. Control eggs (CON) were mock-injected with 0.9% physiological saline. The hole in the eggshell was sealed with a natural glue. The GOS consisted of a formulation of non-digestive mixture of trans-galactooligosaccharides from milk lactose digested with *Bifidobacterium bifidum* [29] (Clasado Biosciences, Jersey, UK). The dose of 3.5 mg/egg of GOS used in this study was chosen on the basis of a previous dose optimization trial, which showed that this dose does not reduce the hatchability rate [30].

At hatching, chicks were sexed and vaccinated against coccidiosis, Infectious Bronchitis Virus, Marek’s disease virus, Newcastle and Gumboro disease. Hatchability was calculated based on the proportion of fertile to hatched eggs (candling was done on day 12 of egg incubation). A total of 300 male chicks/group (i.e., CON and GOS) were transferred to the experimental facility of the University of Bologna within an environmentally controlled poultry house. In the poultry house, the 300 chicks belonging to each group were divided into two subgroups (150 chicks each). Each subgroup was split into 6 replicates (25 birds/replicate). Pens were distributed in randomized block within the poultry facility to mitigate possible environmental effect. Chickens were kept in thermal conditions respective to age. On days 32–42 chronic heat stress (HS) was applied by increasing ambient temperature to 30 °C. Control animals were kept in thermoneutral (TN) conditions (25 °C) (all birds received the same standard commercial diet composed by three feeding phases: starter (0–10 d), grower (11–25 d) and finisher (26–42 d)) (Table S1). More details and the results of the growth performance are reported in another paper [27], where two negative control groups (no *in ovo* treatment in thermoneutral or heat stress condition) were present. These two groups were excluded from the present experiment because assessing the general effect of the *in ovo* treatment on the intestinal transcriptome and immunity was not an aim.

On day 42, 12 birds/group were selected from each treatment group (2 birds per pen, those with BW closer to the pen mean). Jejunum and cecum mucosae were collected by gently scraping after rinsing tissues in PBS to remove residues of digesta and were immediately frozen in liquid nitrogen and stored at −80 °C. At the same time, blood was collected from the wing vein from 2 birds/replicate using 6 mL EDTA coated vacutainer tubes (Vacumed K3 EDTA, vacuum system). Immediately after sampling, tubes were centrifuged at 4000× *g* for 15 min at 4 °C. Then, plasma was quickly dispensed in vials, snap-frozen in liquid nitrogen and stored at −80 °C.

### 2.2. RNA and Plasma Analysis

Total RNA was extracted from jejunum and cecum mucosae using GeneJET RNA Purification Kit (Thermo Scientific, Waltham, MA, USA) according to manufacturer’s instructions. RNA quantity and quality were evaluated using Nanodrop ND 1000 spectrophotometer (Nanodrop Technologies Inc., Wilmington, DE, USA) and agarose gel electrophoresis. The RNA integrity was evaluated through Agilent Bioanalyzer 2100 (Agilent Technologies, Santa Clara, CA, USA). The whole transcriptome microarray analysis was performed on a total of 40 samples (equally distributed between the two tissue, the two diets and the two environmental condition) using Affimetrix© GeneChip Chicken Gene 1.0 ST Array. Hybridized arrays were scanned on Affimetrix© GeneChip Scanner 3000 7G System (Affymetrix, Santa Clara, CA, USA).

Plasma immunoglobulin (Ig) A (IgA), IgG and serum amyloid A (SAA) concentrations were measured according to the protocol of the commercial chicken-specific IgA (Catalogue number ECH0083), IgG (Catalogue number ECH0031) and SAA (Catalogue number ECH0090) Fn-test ELISA kits (Wuhan Fine Biotech Co., Ltd., Wuhan, China). The samples were examined in duplicate at dilutions of 1:1, 1:100000 and 1:4. The IgA, IgG and SAA concentrations of the samples were interpolated from the standard curves using software Curve Expert, version 1.3.

### 2.3. Data Analysis

Transcriptome data were analyzed using Transcriptomic Analysis Console (TAC) Affymetrix© software (4.0.1.36) (Individual records are in the Supplementary Materials—Supplementary 1: Data Base Microarray values). Transcripts were considered as differentially expressed genes (DEG) when showing a ≥2-fold change (log_2_ ratio) and a False Discovery Rate (FDR) < 0.05. TAC was used to test by two-way ANOVA analysis the effect of the interaction between treatment environmental conditions (TN or HS) and *in ovo* injection (CON or GOS) in each tissue. Furthermore, an exploratory functional analysis was then carried out using Gene Set Enrichment Analysis (GSEA) software, which performs a gene set analysis, where gene sets are defined as groups of genes with common biological functions, chromosomal locations or regulation [31]. GSEA analysis was based on C2.CP: KEGG gene set collection (MSigDB, Broad Institute, Cambridge, MA, USA) and gene sets were considered significantly enriched with FDR (q-value) ≤ 0.05. Finally, to evaluate differences between jejunum and cecum within each treatment (i.e., GOS vs. CON and HS vs. TN), the Enrichment Map (http://baderlab.org/Software/EnrichmentMap20) plugin for Cytoscape 3.2.1 (http://www.cytoscape.org) was used in order to visualize the overlap of gene sets, considering a FDR q-value < 0.01. The nodes were joined if the overlap coefficient was ≥ 0.5.

Results from plasma IgA, IgG and SAA were analyzed by two-way ANOVA with R software (Stats Package) considering environmental condition (TN or HS) and *in ovo* injection (CON or GOS) as factors and considering differences statistically significant at *p* ≤ 0.05.

## 3. Results

### 3.1. Transcriptomic Profile

No interaction between environmental conditions (i.e., TN and HS) and *in ovo* treatments (CON vs. GOS) was detected in jejunum and cecum mucosa for differentially expressed genes (DEGs); therefore, the effects of heat stress and *in ovo* treatment were evaluated separately. Considering the GOS vs. CON treatment, no single DEG was detected in either tissue. Conversely, HS vs. TN up- and down-regulated 11 and 13 single genes in jejunum and 2 and 9 single genes in cecum, respectively, considering FDR < 0.05 (Tables S2 and S3). 

Gene set analysis using GSEA was carried out on all the transcriptome data in order to evidence whether the interaction among genes determined the presence of significantly different gene sets in the tissues and treatments involved. Indeed, GSEA analysis highlighted significant associations of genes corresponding to different biological pathways activated; single-gene analysis may have been a more limited approach [31]. In jejunum mucosa, 11 significantly enriched gene sets were observed in the GOS group, mainly linked to energetic metabolism and oxidation (PEROXISOME, SPHINGOLIPID METABOLISM, CYTOCHROME P450 METABOLISM, PENTOSE-PHOSPHATE PATHWAY, FATTY ACID METABOLISM; FDR ≤ 0.008), while in the CON group, 13 enriched gene sets were detected, including CELL CYCLE, DNA REPLICATION and RIBOSOME (FDR ≤ 0.002) as the first three enriched gene sets (Table 1). In cecum mucosa, 11 enriched gene sets were observed in the CON group, most of them being grouped and linked to immune cell response (PHOSPHATIDYLINOSITOL SIGNALING SYSTEM, T CELL RECEPTOR SIGNALING PATHWAY; B CELL RECEPTOR SIGNALING PATHWAY; CHEMOKINE SIGNALING PATHWAY; NATURAL KILLER CELL MEDIATED CYTOTOXICITY; FDR ≤ 0.028); only one gene set was enriched in the GOS group for cecum (ECM RECEPTOR INTERACTION) (Table 2).

For HS, the jejunum GSEA analysis showed enrichment in four gene sets, including METABOLISM CYTOCHROME P450 and METABOLISM OF XENOBIOTICS BY CYTOCHROME P450 (FDR ≤ 0.001), while 14 enriched gene sets were found in the TN group, among which were OXIDATIVE PHOSPHORYLATION as the first gene set and immune-related gene sets (INTESTINAL IMMUNE NETWORK FOR IGA PRODUCTION, CHEMOKINE SIGNALING PATHWAY, B CELL RECEPTOR SIGNALING PATHWAY; FDR ≤ 0.016) were observed (Table 3). In cecum mucosa, seven enriched gene sets were found in the HS group, including ECM RECEPTOR INTERACTION and DNA REPLICATION, while 27 gene sets were enriched in the TN group, including gene sets linked to energetic metabolism (SPHINGOLIPID METABOLISM, STARCH AND SUCROSE METABOLISM, OXIDATIVE PHOSPHORYLATION; FDR = 0.000) and immune response (NATURAL KILLER CELL MEDIATED CYTOTOXICITY, B CELL RECEPTOR SIGNALING PATHWAY, T CELL RECEPTOR SIGNALING PATHWAY; FDR ≤ 0.001) (Table 4).

**Figure 1:**
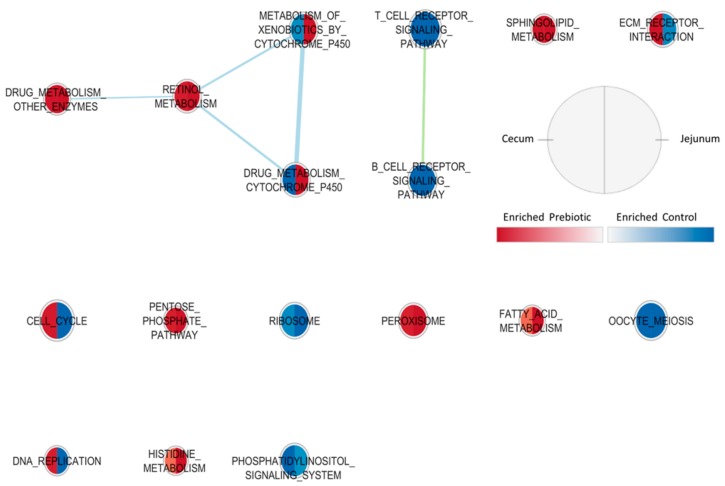
Enrichment Map showing enriched gene sets in cecum and jejunum of broiler chickens (42 days of age) *in ovo* injected with a single dose of physiological saline (0.2 mL of 0.9% NaCl), control (CON), or 0.2 mL of 0.9% physiological saline + 3.5 mg galactooligosaccharide prebiotic/egg, prebiotic (GOS) (n = 20 per treatment). Nodes represent gene sets. Each gene set enrichment is represented for both cecum (left side of the area) and jejunum (right side of the area) tissue, enriched or in the prebiotic group (red color) or in the control group (blue color). The node size represents the number of genes in each gene set. Node cut off with FDR q-value < 0.01. Nodes were joined if the overlap coefficient was ≥0.5.

**Figure 2:**
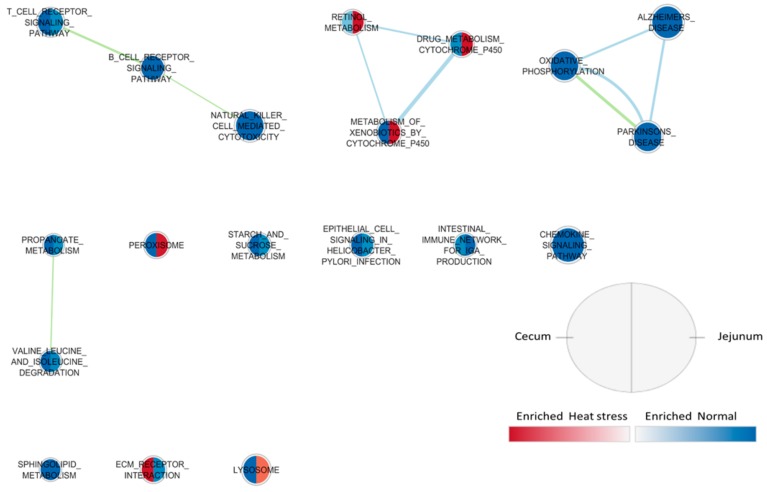
Enrichment Map showing enriched gene sets in cecum and jejunum of broilers (42 days of age) *in ovo* injected, in thermoneutral (normal) (TN, 25 °C) and heat stress (HS, 30 °C constantly) groups (n = 20 per treatment). Nodes represent gene sets. Each gene set enrichment is represented for both cecum (left side of the area) and jejunum (right side of the area) tissue, enriched or in heat stress group (red color) or in normal (thermoneutral) group (blue color). Node size represents the number of genes in each gene set. Node cut off with FDR q-value <0.01. The nodes were joined if the overlap coefficient was ≥0.5.

Results from the Enrichement Map for gene sets enriched in cecum and jejunum of broiler chicken *in ovo* injected with GOS or CON are graphically presented in Figure 1. In both tissues, enriched gene sets regarding immune cell signaling pathways were found as up-regulated in the CON group, while gene sets such as PENTOSE-PHOSPHATE PATHWAYS, FATTY ACID METABOLISM AND PEROXISOME were generally up-regulated in the GOS group. Other gene sets related to metabolic processes (gene sets regarding metabolism by cytochrome p450) were found enriched in GOS, specifically in jejunum. These results confirm the GSEA results where, with GOS, a general up-regulation of gene sets related to energy metabolism was reported, while in CON gene sets related to immunity were highlighted. In Figure 2, results from the Enrichement Map are displayed considering gene sets enriched in cecum and jejunum mucosa of broilers *in ovo* injected and submitted to TN or HS conditions. Considering the heat stress treatment, gene sets were enriched in a homogeneous way in the two tissues: Most of gene sets, such as OXIDATIVE PHOSPHORYLATION, STARCH AND SUCROSE METABOLISM, and amino acid metabolism and immune response-related gene sets, were enriched in the TN group.

### 3.2. Plasma IgG, IgA, SAA

No interaction between environmental conditions and *in ovo* treatments was detected for serum IgG, IgA and SAA levels. No significant differences were observed in serum immune parameters between environmental treatments and between *in ovo* injection treatments (Table 5).

## 4. Discussion

### 4.1. Transcriptome

#### 4.1.1. GOS and Gene Sets Related to Immune Response

Based on the transcriptomic profile of the studied tissues, the GOS probiotic administration overall was seen to reduce function related to inflammatory signaling, and the effect was greater in the gene sets from the CON group in cecum compared to all groups in jejunum.

In fact, in cecum, the PHOSPHATIDYLINOSITOL SIGNALING SYSTEM gene set was enriched in CON and not in the GOS group. Among the genes in this gene set, *PIK3C2B* (a gene belonging to PI3K phosphatidylinositol-phosphate 3 kinase family, involved in signaling pathway for cell proliferation, migration, etc.) was one of the most significant defining the gene set, and it was up-regulated in the CON group. The expression of *PI3K* is reported to be positively correlated with toll-like receptors (*TLRs*) gene expression, which, in turn, is up-regulated by cytokines in inflammatory conditions [32]. *MAP2K2* (belonging to MAPK family) was the second and third gene we found, respectively, in the other immune-related gene sets, T CELL RECEPTOR SIGNALING PATHWAY and B CELL RECEPTOR SIGNALING PATHWAY. According to literature, the phosphorylation of *PI3K* and *MAPK* (mitogen-activated protein kinase family), induced by TLRs signaling, leads to NF-κB (nuclear factor kappa-light-chain-enhancer of activated B cells) activation, which acts as a transcription factor for immune response and as stimulator for pro-inflammatory cytokines production [32]. This suggests it has a possible correlation and close role with *PIK3C2B* (in PHOSPHATIDYLINOSITOL SIGNALING SYSTEM gene set) in immune system stimulation. In support of this, *CD8 T* cell marker gene was also found as the third and first gene listed in T CELL RECEPTOR SIGNALING PATHWAY and B CELL RECEPTOR SIGNALING PATHWAY gene set, respectively. Indeed, CD8 T cells can secrete different cytokines, have pro-inflammatory functions, and affect B cell response [33].

Moreover, the CON group in cecum up-regulated other gene sets related to immunity and cytokines: CHEMOKINE SIGNALING PATHWAY and NATURAL KILLER CELL MEDIATED CYOTOTOXICITY. It is worth noting that the release of cytokines by immune cells is negatively correlated with the cytochrome p450 (CYP) drug/xenobiotic metabolic capacity, meaning that a decreased metabolic activity could be associated with inflammatory status [34].

It is important to consider that T CELL RECEPTOR SIGNALING PATHWAY and B CELL RECEPTOR SIGNALING PATHWAY gene sets were up-regulated in the CON group compared to GOS (this was also the case in jejunum), demonstrating that in this tissue the CON group showed the activation of pathways related to the inflammation process.

On the contrary, the GOS group in jejunum showed expression of the DRUG METABOLISM CYTOCHROME P450 and METABOLISM OF XENOBIOTICS BY CYTOCHROME P450 gene sets. This might be the effect of GOS administration, since a similar effect was observed in response to drug administration under inflammatory condition, that was found to induce the down-regulation of hepatic and extrahepatic CYP enzymes [35]. It is worth nothing that small intestinal mucosa is the most important extrahepatic site of biotransformation where CYP enzymes play a key role in metabolic processes [34,36]. These results indicate gene and function similarities between human and chicken at the small intestinal level. Since intestinal metabolic processes mainly occur in the small intestine, it might be possible that CYP metabolism-related gene sets were poorly enriched in cecum. In both of these gene sets, *CYP2D6* (xenobiotic detoxifying CYP enzyme) and *UGT2A1* (xenobiotic/endobiotic compound-metabolizing enzyme of UDP glucuronosyltransferase 2 family) genes were found among the first genes of the list. A similar *UDP* glucuronosyltransferase, *UGT1A1* (drug-metabolizing enzyme involved in gut epithelial barrier maintenance) was investigated by Gao et al. [32]. They observed a decrease of gut UGT1A1 protein concentration in rats with colitis, confirming the negative correlation between metabolic capacity and inflammation. After, they also reported a decrease of *UGT1A1* gene expression in a condition of gut dysbiosis induced by Gram-negative bacteria, both in normal and colitis rats, showing a possible relevant role of microbiota in xenobiotic metabolizing enzymes expression regulation [32]. An enriched gene set for retinol metabolism was also found in GOS group, mainly in jejunum, but was also highlighted in both tissues by the Enrichment Map, and, similar to the CYP DRUG METABOLISM gene set, the first genes of the list were related to xenobiotic metabolism (*UGT2A1* and *CYP3A7*). Considering these observations, microbial population might have developed differently in CON and GOS, as observed by Slawinska et al. [37], leading to a different immune stimulation. A different microbiota might have led to a higher immune defense recruitment in the CON group compared to the GOS group, where, particularly in jejunum, the enriched gene set of CYP metabolic enzymes might be related to a better gut function, since the *CYP* intestinal role also concerns endogenous metabolism, such as that for fatty acids [36], and a good epithelial barrier with less expenditure in immune system stimulation can lead to a greater energy saving. 

#### 4.1.2. GOS and Gene Sets Related to Energy Metabolism

Another functional group significantly enriched by GSEA analysis concerned genes involved in energetic metabolism.

In particular, the enriched gene set PENTOSE-PHOSPHATE PATHWAY was found to be higher in the GOS group, specifically in jejunum, meaning there was a higher energetic metabolism in this group. Some of the genes found to be more significantly enriched were those coding for enzymes involved in glucose metabolism, such as *FBP1* (fructose 1,6 biphosphatase 1) and *PFKL* (phosphofructokinase), and for enzymes involved in ribose metabolism (*RBKS*, ribokinase). The hypothesis of a higher energetic metabolism in GOS may be linked to the presence of the enriched PEROXISOME gene set. Peroxisomes are pivotal to several lipid-metabolizing pathways [38]. In fact, inside the gene set in jejunum, at the top of the list there was *ACOX2* gene (acylCoA oxidase 2, involved in branched fatty acid degradation), along with *EHHADH* gene (encoding for a beta oxidation pathway enzyme); both are key enzymes for beta oxidation also found in mammal small intestines [38], and *CAT* gene (catalase, H_2_O_2_ detoxifying enzyme and peroxisomal marker). The analysis with Enrichment Maps showed that the PENTOSE-PHOSPHATE PATHWAY and PEROXISOME gene sets were also enriched in the GOS group in cecum, leading us to hypothesize that the effect was smaller in cecum than in jejunum, as reported by Morvay et al. [38], who found peroxisomes to be mainly present in the small rather than large intestine of mice, as well as the higher expression of *CAT* and *ACOX2* genes, due to higher involvement of small intestine in lipid uptake [38]. Then, peroxisome involvement in metabolic oxidation processes, in turn, can be linked to the enrichment of FATTY ACID METABOLISM gene set in jejunum, where again *EHHADH* was one of the genes at the top of the list, along with *ALDH3A2* (aldehyde dehydrogenase, detoxifying aldehydes from lipid peroxidation) and *ACOX1*. In support of this hypothesis, we also found PPARs SIGNALING PATHWAY gene set in jejunum. This pathway regulates peroxisomal beta oxidation of fatty acids (and the gene *ACOX2* found in PEROXISOME gene set was also in this list), along with retinoid receptors. *PPARs* are also upstream regulators of *UDP* glucuronosyltransferases (involved in xenobiotics metabolism) such as *UGT1A1*, as reported by [32], who also hypothesized that microbiota might regulate these receptors in the gut. The SPHINGOLIPID METABOLISM gene set was also enriched in GOS, in jejunum firstly, but was shown to be enriched in both tissues in the Enrichment Map. Beneficial bacteria like *Bacteroides* can produce sphingolipids and these molecules are involved in bacterial–host interactions in immune system modulation and in gut homeostasis maintenance, acting as signal molecules [39]. Therefore, it might be possible that in the GOS group, a different microbiota (compared to CON group) influenced the lipidic metabolic pathways and that a higher sphingolipid metabolism could have contributed to gut function and barrier maintenance. Stimulation of genes related to the SPHINGOLIPID METABOLIC PROCESSES gene set was previously found also in pig jejunal loops perfused in vivo with a starter microbiota [40]. As a final hypothesis, a less activated immune system along with a high energetic metabolism could have explained an increased growth efficiency of the GOS group compared to the CON group. However, in the second period of growth, GOS were actually more efficient, but only against the groups that were not *in ovo* injected, which were not analyzed for the present trial, while there was only a numerical decrease of feed conversion ratio against the CON group [30]. Some discrepancies between the observations on the intestinal transcriptome and the overall performance can be explained by the fact that the first is a picture taken at a specific time (the moment of the slaughtering), while the performance efficiency is the sum of the animal metabolic adjustments over a period of 14 days.

From GSEA analysis, only one gene set was enriched in cecum in the GOS group, that is, ECM RECEPTOR INTERACTION. Considering the ECM role in structurally supporting gut mucosa, it is possible that GOS led to an improvement in barrier maintenance in cecum. This hypothesis may be supported by the gene sets found in the Enrichment Map. In fact, the CELL CYCLE and DNA REPLICATION gene sets were enriched in cecum when compared to jejunum, suggesting more cell turnover, which may be involved in gut barrier maintenance. Moreover, results of a study on microbiota revealed that there is a possible bifidogenic effect of GOS on butyric-producing bacteria, and butyrate is used as a main energetic source in cecum [41], suggesting that a different microbiome in the gut may lead to different biological pathways activation. Further studies are deemed necessary to confirm this hypothesis. 

#### 4.1.3. Heat Stress Effect

As expected, HS strongly affected gene set enrichment. Indeed, enrichment analysis showed fewer enriched gene sets in the HS group compared to the TN group. Indeed, the TN group showed an up-regulation of gene sets related to oxidative phosphorylation, amino acid metabolism (VALINE, LEUCINE AND ISOLEUCINE DEGRADATION) and immune response (T CELL AND B CELL RECEPTOR SIGNALING PATHWAY; NATURAL KILLER CELL MEDIATED CYTOTOXICITY). As already reported from the literature, heat stress leads to mitochondrial damage (since mitochondria are the main responsible for reactive oxygen species—ROS production, in case of oxidative stress), and their damage leads to inactivation of the respiratory chain, decreasing cellular energy production following the alteration of oxidative phosphorylation pathway [42]. Furthermore, down-regulation of cellular energetic metabolism might be linked to another gene set found to be up-regulated in the TN group compared to the HS group, that is, STARCH AND SUCROSE METABOLISM. Chronic HS seems to negatively affect protein metabolism [42]. In fact, we found amino acid metabolism-related gene sets were downgraded in the HS group compared to the TN group, with the gene *ACAD8* (acylCoA dehydrogenase family member 8) at the top of the list. *ACAD8* encodes for a dehydrogenase involved in branched-chain amino acids (*BCAAs*) metabolism. This enzyme is a mitochondrial enzyme and its functionality might be affected by the oxidative stress damage at the mitochondrial level, and especially by chronic heat stress, along with the general decrease of protein breakdown [42]. The downregulation of immune response-related gene sets found in the HS group is in line with what was already reported on poultry, where heat stress induces a general immunosuppression [43], compared to TN conditions. In the TN group, for example, the gene *PIK3R2* (a lipid kinase of the PI3K family, involved in phosphatidylinositol phosphorylation in growth signaling pathway and in activation of NF-κB complex inducing immune response) was found to be one of the key genes in all three gene sets (T CELL AND B CELL RECEPTOR SIGNALING PATHWAY; NATURAL KILLER CELL MEDIATED CYTOTOXICITY).

Immunosuppression in the HS group might be linked to a cortisol effect or can also be a consequence of the lower feed intake that occurred at high temperatures, as shown by Slawinska et al. [30]. Both events could have produced a general adjustment of metabolic activity in the cells. Accordingly, as reported by Slimen et al. [42], growth and health are not priorities in the metabolism of heat-stressed animals, due to their lower metabolic rates [42]. Nevertheless, these adjustment in were not sufficient to maintain the same energetic efficiency of the TN group because the feed efficiency was impaired in the HS group during the period of heat stress [30]. This could have been because of the increased relative incidence of energy used for maintenance costs.

Finally, results from the Enrichment Map for the enriched gene sets were similar between cecum and jejunum tissues belonging to the HS group. This result indicates that, despite possible variations between tissues because of local microbiota, the two intestinal tracts react similarly at the molecular level when subjected to heat stress.

### 4.2. Plasma IgG, IgA, SAA

Regarding results on serum immune parameters IgG, IgA and SAA (Table 5), no differences were found between the environmental and *in ovo* treatments.

IgG is the major class of blood circulating antibodies produced in the humoral response to neutralize antigens and activate macrophages and the complement system [44]. Some previous studies reported IgG decrease as a marker of heat stress-induced immunosuppression [45,46]. However, these authors observed IgG suppression following extreme heat stress conditions (over 33 °C), so it is probable that the heat stress induced in our study did not stimulate IgG levels in the same way.

Concerning IgA concentrations, it may be possible that, since this Ig class is found primarily at the intestinal level, only a strong impairment of the gut barrier may provoke high IgA serum levels due to a damaged mucosal layer and to exposition of antigens [47], which probably did not happen in our case. However, in contrast with previous results, a more recent trial reported that IgG and IgA significantly increased in broiler chickens under chronic heat stress [48]. Since Ig belong to serum non-specific molecules released in different contexts of immune responses and in response to different inflammatory processes, many factors may affect their trend in serum. Our results regarding serum IgG levels in the sGOS or CON group are in line with results reported by Midilli et al. [49], where dietary prebiotic (0.2%) supplementation in broiler chicken did not affect IgG serum levels at d 42 [49]. Conversely, in a study on turkeys it was reported that IgG values were higher in the group fed dietary mannooligosaccharide (0.5%, MOS) than in the CON group [50]. Regarding IgA, as we observed in this study, Kim et al. [51] also did not find differences in plasma IgA levels in broiler fed 0.5% dietary prebiotic fructo-oligosaccharides (FOS) or MOS at d 21 [51]. In contrast, Rezaei et al. [52] found increased blood IgA levels in broiler fed 1% palm kernel oligosaccharide prebiotic at d 36. Possible explanations for the different results may refer to the doses and to the different delivery methods of prebiotics (orally or *in ovo*). 

Regarding SAA levels, our results contrast with those found by Hartog et al. [53], where a dietary prebiotic (1.5% prebiotic multifiber mixture) significantly reduced SAA serum levels in mice with induced colitis [53]. Other previous studies reported significant increases in serum SAA levels in chicks infected with bronchitis virus [54,55] and in chicken vaccinated against infectious bronchitis virus and Newcastle disease [56], where a strong stimulation of immunity against infections occurs. Even if SAA is a phase-acute protein proposed to be a general marker of stress, it might be possible that the SAA concentration in serum changes depending on the inflammation type and stress level, and so is related to the severity of stimulation or damage.

## 5. Conclusions

A general favorable effect of GOS prebiotic may be recognized due to the enrichment of energetic metabolism-related gene sets, mainly in jejunum. The enrichment of lipidic metabolism-related gene sets in the GOS group might have contributed to gut function and barrier maintenance, which might also be linked to reduced immune system activation, mainly at the cecum level. Considering HS, the experimental model was effective in stressing the animals, according to previous studies, with impairment of gut functions in terms of energy and immunity. Generally, our results showed that the additional efficacy of GOS on transcriptome in the case of heat stress was negligible. Nevertheless, without considering the different environmental conditions, the positive impact of GOS on transcriptome data concurs to sustain the ability of the *in ovo* injection technique to induce long-term positive effects, confirming this strategy as a tool to modify the early programming development of the chick gut.

## Figures and Tables

**Table 1:** Enriched gene sets found in jejunum mucosa of broiler chickens (42 days of age), after GSEA analysis considering FDR q value ≤ 0.05 (n = 20 per in ovo treatment).

Gene Set—Jejunum GOS ^1^	FDR q Value
PEROXISOME	0.000
SPHINGOLIPID METABOLISM	0.000
HISTIDINE METABOLISM	0.001
DRUG METABOLISM CYTOCHROME P450	0.007
METABOLISM OF XENOBIOTICS BY CYTOCHROME P450	0.006
DRUG METABOLISM OTHER ENZYMES	0.006
PENTOSE PHOSPHATE PATHWAY	0.006
FATTY ACID METABOLISM	0.008
RETINOL METABOLISM	0.008
STARCH AND SUCROSE METABOLISM	0.011
PPAR SIGNALING PATHWAY	0.013
**Gene Set—Jejunum CON ^2^**	**FDR q Value**
CELL CYCLE	0.000
DNA REPLICATION	0.000
RIBOSOME	0.002
OOCYTE MEIOSIS	0.006
SYSTEMIC LUPUS ERYTHEMATOSUS	0.014
SPLICEOSOME	0.016
PROGESTERONE MEDIATED OOCYTE MATURATION	0.016
B CELL RECEPTOR SGNALING PATHWAY	0.020
MISMATCH REPAIR	0.024
HOMOLOGOUS RECOMBINATION	0.030
NUCLEOTIDE EXCISION REPAIR	0.034
T CELL RECEPTOR SIGNALING PATHWAY	0.033
PROTEASOME	0.050

^1^ Gene Set—Jejunum GOS: GOS group (0.2 mL of 0.9% physiological saline + 3.5 mg prebiotic (GOS)/egg) vs. CON (0.2 mL of 0.9% physiological saline); ^2^ Gene Set—Jejunum CON: CON group (0.2 mL of 0.9% physiological saline) vs. GOS group (0.2 mL of 0.9% physiological saline + 3.5 mg prebiotic (GOS)/egg.

**Table 2:** Enriched gene sets found in cecum mucosa of broiler chickens (42 days of age), after GSEA analysis considering FDR q value ≤ 0.05 (n = 20 per *in ovo* treatment).

Gene Set—Cecum GOS ^1^	FDR q Value
ECM RECEPTOR INTERACTION	0.008
**Gene Set—Cecum CON ^2^**	**FDR q Value**
PHOSPHATIDYLINOSITOL SIGNALING SYSTEM	0.000
T CELL RECEPTOR SIGNALING PATHWAY	0.000
B CELL RECEPTOR SIGNALING PATHWAY	0.009
GLIOMA	0.015
FC GAMMA R MEDIATED PHAGOCYTOSIS	0.016
RIG I LIKE RECEPTOR SIGNALING PATHWAY	0.017
CHEMOKINE SIGNALING PATHWAY	0.015
ENDOCYTOSIS	0.018
NATURAL KILLER CELL MEDIATED CYTOTOXICITY	0.028
UIBQUITIN MEDIATED PROTEOLYSIS	0.035
INOSITOL PHOSPHATE METABOLISM	0.034

^1^ Gene Set—Cecum GOS: GOS group (0.2 mL of 9% physiological saline + 3.5 mg prebiotic (GOS)/egg vs. CON group (0.2 mL of 0.9% physiological saline); ^2^ Gene Set—Cecum CON: CON group (0.2 mL of 0.9% physiological saline) vs. GOS group (0.2 mL of 9% physiological saline + 3.5 mg prebiotic (GOS)/egg.

**Table 3:** Enriched gene sets found in jejunum mucosa of broiler chickens (42 days of age), after GSEA analysis considering FDR q value ≤ 0.05 (n = 20 per thermal treatment).

Gene Set—Jejunum HS ^1^	FDR q Value
DRUG METABOLISM CYTOCHROME P450	0.001
METABOLISM OF XENOBIOTICS BY CYTOCHROME P450	0.001
RETINOL METABOLISM	0.004
BASAL TRANSCRIPTION FACTORS	0.034
**Gene Set—Jejunum TN ^2^**	**FDR q Value**
OXIDATIVE PHOSPHORYLATION	0.000
PARKINSONS DISEASE	0.000
ALZHEIMERS DISEASES	0.000
PROTEASOME	0.011
INTESTINAL IMMUNE NETWORK FOR IGA PRODUCTION	0.010
CHEMOKINE SIGNALING PATHWAY	0.008
CELL ADHESION MOLECULES CAMS	0.015
B CELL RECEPTOR SIGNALING PATHWAY	0.016
HUNTINGTONS DISEASE	0.021
FC EPSILON RI SIGNALING PATHWAY	0.020
AMINO SUGAR AND NUCLEOTIDE SUGAR METABOLISM	0.030
PROTEIN EXPORT	0.040
OLFACTORY TRANSDUCTION	0.044
N GLYCAN BYOSINTHESIS	0.046

^1^ Gene Set—Jejunum HS: HS group (HS—heat stress, 30 °C for 24h/d from 32 to 42 d) vs. TN group (TN—thermoneutral, 25 °C); ^2^ Gene Set—Jejunum TN: TN group (TN—thermoneutral, 25 °C) vs. HS group (HS—heat stress, 30 °C for 24h/d from 32 to 42 d).

**Table 4:** Main gene sets found in cecum mucosa of broiler chickens (42 days of age), after GSEA analysis considering FDR q value ≤ 0.05 (n = 20 per thermal treatment).

Gene Set—Cecum HS ^1^	FDR q Value
ECM RECEPTOR INTERACTION	0.001
DNA REPLICATION	0.029
SPLICEOSOME	0.062
RNA POLYMERASE	0.047
COMPLEMENT AND COAGULATION CASCADES	0.042
BASAL CELL CARCINOMA	0.035
SYSTEMIC LUPUS ERYTHEMATOSUS	0.042
**Gene Set—Cecum TN ^2^**	**FDR q Value**
PEROXISOME	0.000
NATURAL KILLER CELL MEDIATED CYTOTOXICITY	0.000
SPHINGOLIPID METABOLISM	0.000
STARCH AND SUCROSE METABOLISM	0.000
PROPANOATE METABOLISM	0.000
OXIDATIVE PHOSPHORYLATION	0.000
B CELL RECEPTOR SIGNALING PATHWAY	0.000
LYSOSOME	0.000
T CELL RECEPTOR SIGNALING PATHWAY	0.001
PARKINSONS DISEASE	0.005
EPITHELIAL CELL SIGNALING IN HELICOBACTER PYLORI INFECTION	0.008
VALINE LEUCINE AND ISOLEUCINE DEGRADATION	0.008
ALDOSTERONE REGULATED SODIUM REABSORPTION	0.011
ALZHEIMERS DISEASE	0.011
UBIQUITIN MEDIATED PROTEOLYSIS	0.023
CHEMOKINE SIGNALING PATHWAY	0.022
INSULIN SIGNALING PATHWAY	0.022
FC EPSILON RI SIGNALING PATHWAY	0.025
ENDOCYTOSIS	0.031
VIBRIO CHOLERAE INFECTION	0.036
TOLL-LIKE RECEPTOR SIGNALING PATHWAY	0.037
CITRATE CYCLE TCA CYCLE	0.038
FC GAMMA R MEDIATED PHAGOCYTOSIS	0.039
PPAR SIGNALING PATHWAY	0.039
FATTY ACID METABOLISM	0.040
BUTANOATE METABOLISM	0.042
GALACTOSE METABOLISM	0.046

^1^ Gene Set—Cecum HS: HS group (HS—heat stress, 30 °C for 24h/d from 32 to 42 d) vs. TN group (TN—thermoneutral, 25 °C); ^2^ Gene Set—Cecum TN: TN group (TN—thermoneutral, 25 °C) vs. HS group (HS—heat stress, 30 °C for 24h/d from 32 to 42 d).

**Table 5:** Plasma immune parameters of broiler chickens at 42 days of age with *in ovo* (CON group, 0.2 mL of 0.9% physiological saline, vs. GOS group, 0.2 mL of 9% physiological saline + 3.5 mg prebiotic GOS/egg, n = 24 per treatment) and thermal (TN—thermoneutral, 25 °C, vs. HS—heat stress, 30 °C for 24h/d from 32 to 42 d, n = 24 per treatment) treatments as factors.

Items	*In ovo* Treatment				Environmental Treatment				*p* Value		
	GOS	SEM	CON	SEM	HS	SEM	TN	SEM	*In ovo*	Environmental	Interaction
IgG mg/mL	4.43	0.46	4.82	0.54	4.61	0.55	4.63	0.44	0.58	0.97	0.30
IgA ng/mL	25.01	8.21	34.45	4.65	30.50	7.21	28.40	6.68	0.36	0.87	0.57
SAA ng/mL	4.16	0.62	4.04	0.38	4.24	0.64	3.95	0.36	0.88	0.70	0.65

Immunoglobulin G (IgG); Immunoglobulin A (IgA); serum amyloid A (SAA). Differences are considered significant with *p* < 0.05. Values are reported as the mean and standard error of the mean (SEM).

## Supplementary Materials

The following are available online at https://www.mdpi.com/2076-2615/9/12/1067/s1, Supplementary 1: Data Base Microarray values, Table S1: Composition of the commercial diets, Table S2: Differentially expressed genes found in jejunum mucosa of broiler chickens (42 days of age) of HS group (HS—heat stress, 30 °C for 24 h/d from 32 to 42 d) vs. TN group (TN—thermoneutral, 25 °C), considering a ≥2-Fold Change (log_2_ ratio) and FDR q value ≤0.05 (n = 20 per thermal treatment), Table S3: Differentially expressed genes found in cecum mucosa of broiler chickens (42 days of age) of HS group (HS—heat stress, 30 °C for 24 h/d from 32 to 42 d) vs. TN group (TN—thermoneutral, 25 °C), considering a ≥2-Fold Change (log_2_ ratio) and FDR q value ≤0.05 (n = 20 per thermal treatment).
