# Supplementary material for: In ovo Injection of a Galacto-Oligosaccharide Prebiotic in Broiler Chickens Submitted to Heat-Stress: Impact on Transcriptomic Profile and Plasma Immune Parameters
Source: Animals (Basel). 2019 Dec 2;9(12):1067. doi: 10.3390/ani9121067 (PMC6940861; doi:10.3390/ani9121067)
Supplement: Supplementary file 1 [file animals-09-01067-s001.zip › animals-653864-ffsup/Supplementary Tables.docx]

**Table S1.** Composition of the commercial diets.

| Item | STARTER (0–10 d) | GROWER 1 (11–25 d) | FINISHER (26–42 d) |
| --- | --- | --- | --- |
| Corn | 42.17 | 34.96 | 12.73 |
| White Corn | 0.00 | 0.00 | 15.00 |
| Wheat | 10.00 | 20.00 | 25.01 |
| Sorghum | 0.00 | 0.00 | 5.00 |
| Soybean Meal | 23.11 | 20.63 | 17.60 |
| Expanded Soybean | 10.00 | 10.00 | 13.00 |
| Sunflower | 3.00 | 3.00 | 3.00 |
| Corn Gluten | 4.00 | 3.00 | 0.00 |
| Soybean Oil | 3.08 | 4.43 | 5.48 |
| Dicalcium phosphate | 1.52 | 1.20 | 0.57 |
| Calcium carbonate | 0.91 | 0.65 | 0.52 |
| Sodium bicarbonate | 0.15 | 0.10 | 0.15 |
| Salt | 0.27 | 0.27 | 0.25 |
| Choline chloride | 0.10 | 0.10 | 0.10 |
| Lysine sulphate | 0.59 | 0.55 | 0.46 |
| Dl-Methionine | 0.27 | 0.29 | 0.30 |
| Threonine | 0.15 | 0.14 | 0.14 |
| Enzyme—Roxazyme G2G | 0.08 | 0.08 | 0.08 |
| Phytase 0.1% | 0.10 | 0.10 | 0.10 |
| Vitamin—Mineral Premix ^1^ | 0.50 | 0.50 | 0.50 |
| Dry Matter, % | 88.57 | 88.65 | 88.64 |
| Protein, % | 22.70 | 21.49 | 19.74 |
| Lipid, % | 7.06 | 8.24 | 9.74 |
| Fibre, % | 3.08 | 3.04 | 3.07 |
| Ash, % | 5.85 | 5.17 | 4.49 |
| Lysine, % | 1.38 | 1.29 | 1.21 |
| Methionine, % | 0.67 | 0.62 | 0.59 |
| Methionine + Cysteine, % | 1.03 | 0.97 | 0.91 |
| Calcium, % | 0.91 | 0.80 | 0.59 |
| Phosphate, % | 0.63 | 0.57 | 0.46 |
| Metabolizable Energy (Kcal/Kg) | 3.076 | 3.168 | 3.264 |

^1^ Provided the following per kg of diet: vitamin A (retinyl acetate), 13,000 IU; vitamin D3 (cholecalciferol), 4000 IU; vitamin E (DL-α_tocopheryl acetate), 80 IU; vitamin K (menadione sodium bisulfite), 3 mg; riboflavin, 6.0 mg; pantothenic acid, 6.0 mg; niacin, 20 mg; pyridoxine, 2 mg; folic acid, 0.5 mg; biotin, 0.10 mg; thiamine, 2.5 mg; vitamin B12 20 μg; Mn, 100 mg; Zn, 85 mg; Fe, 30 mg; Cu, 10 mg; I, 1.5 mg; Se, 0.2 mg; ethoxyquin, 100 mg.

**Table S2.** Differentially expressed genes found in jejunum mucosa of broiler chickens (42 days of age) of HS group (HS—heat stress, 30 °C for 24 h/d from 32 to 42 d) vs. TN group (TN—thermoneutral, 25 °C), considering a ≥2-Fold Change (log_2_ ratio) and FDR q value ≤0.05 (n = 20 per thermal treatment).

| **Gene Symbol** | **Description** | **HS (log_2_)** | **TN (log_2_)** | **Fold Change** | **FDR *p*-Value** |
| --- | --- | --- | --- | --- | --- |
| MAT2A | methionine adenosyltransferase 2° | 8.1 | 5.64 | 5.51 | 0.0003 |
| MIR215 | microRNA 215 | 3.97 | 2.38 | 3.02 | 0.0038 |
| FAXDC2 | fatty acid hydroxylase domain containing 2 | 7.76 | 6.23 | 2.88 | 0.0364 |
| ENSGALG00000048005 | LncRNA | 5.9 | 4.39 | 2.86 | 0.0002 |
| MIR141 | microRNA 141 | 4.98 | 3.63 | 2.54 | 0.0021 |
| CHGA | chromogranin A | 9.41 | 8.15 | 2.4 | 0.0002 |
| ENSGALG00000040296 | Mt tRNA | 9.41 | 8.22 | 2.28 | 0.0468 |
| SCN3B | sodium channel, voltage-gated, type III, beta subunit | 5.18 | 4.09 | 2.13 | 0.0005 |
| NDRG1 | N-myc downstream regulated 1 | 9.21 | 8.12 | 2.13 | 0.0064 |
| F3 | coagulation factor III (thromboplastin, tissue factor) | 5.62 | 4.59 | 2.04 | 0.0128 |
| RAB36 | RAB36, member RAS oncogene family | 3.87 | 2.85 | 2.02 | 0.0013 |
| CHDH | choline dehydrogenase | 6.32 | 7.35 | −2.04 | 0.0012 |
| CMAS | cytidine monophosphate N-acetylneuraminic acid synthetase | 6.12 | 7.17 | −2.07 | 0.0002 |
| ASPA | aspartoacylase | 4.82 | 5.93 | −2.16 | 0.0171 |
| ADAMTS18 | ADAM metallopeptidase with thrombospondin type 1 motif, 18 | 3.63 | 4.77 | −2.21 | 0.0005 |
| TMEM252 | transmembrane protein 252 | 7.77 | 8.95 | −2.26 | 0.011 |
| SLC10A2 | solute carrier family 10 (sodium/bile acid cotransporter), member 2 | 4.11 | 5.35 | −2.36 | 0.0028 |
| DNAH14 | dynein, axonemal, heavy chain 14 | 3.35 | 4.65 | −2.46 | 0.0299 |
| RGCC | regulator of cell cycle | 4.52 | 5.95 | −2.69 | 0.0003 |
| SUSD2 | sushi domain containing 2 | 6.18 | 7.64 | −2.74 | 0.0005 |
| SLC51B | solute carrier family 51, beta subunit | 5.7 | 7.24 | −2.92 | 0.0108 |
| SLC5A11 | solute carrier family 5 (sodium/inositol cotransporter), member 11 | 6.28 | 8.34 | −4.18 | 0.0002 |
| FABP6 | fatty acid binding protein 6, ileal | 8.51 | 10.63 | −4.33 | 0.0022 |
| TMIGD1 | transmembrane and immunoglobulin domain containing 1 | 6.55 | 8.83 | −4.86 | 0.0232 |

**Table S3.** Differentially expressed genes found in cecum mucosa of broiler chickens (42 days of age) of HS group (HS—heat stress, 30 °C for 24 h/d from 32 to 42 d) vs. TN group (TN—thermoneutral, 25 °C), considering a ≥2-Fold Change (log_2_ ratio) and FDR q value ≤0.05 (n = 20 per thermal treatment).

| **Gene Symbol** | **Description** | **HS (log_2_)** | **TN (log_2_)** | **Fold Change** | **FDR *p*-Value** |
| --- | --- | --- | --- | --- | --- |
| MAT2A | methionine adenosyltransferase 2A | 6.17 | 4.46 | 3.27 | 0.0249 |
| STEAP2 | STEAP family member 2, metalloreductase | 4.46 | 3.24 | 2.33 | 0.0367 |
| TMC5 | transmembrane channel-like 5 | 6.29 | 7.35 | −2.09 | 0.0105 |
| TAAR1 | trace amine associated receptor 1 | 4.86 | 5.98 | −2.18 | 0.0313 |
| GPR112 | G protein-coupled receptor 112 | 6.8 | 7.99 | −2.29 | 0.0289 |
| ENSGALG00000042151 | uncharacterized | 8.13 | 9.35 | −2.33 | 0.0059 |
| VNN | vanin 1 | 5.29 | 6.56 | −2.41 | 0.0038 |
| ENSGALG00000035914 | IgGFc-binding protein-like | 8.66 | 10.01 | −2.55 | 0.0298 |
| ENSGALG00000003466 | aldo-keto reductase family 1, member B1-like | 7.86 | 9.23 | −2.57 | 0.0346 |
| CYP51A1 | cytochrome P450, family 51, subfamily A, polypeptide 1 | 6.83 | 8.22 | −2.62 | 0.0277 |
| CALB1 | calbindin 1, 28kDa | 6.02 | 7.92 | −3.73 | 0.0105 |
